# Regulation of toll-like receptors expression in muscle cells by exercise-induced stress

**DOI:** 10.5713/ab.20.0484

**Published:** 2020-12-01

**Authors:** Jeong-Woong Park, Kyung-Hwan Kim, Joong-Kook Choi, Tae Sub Park, Ki-Duk Song, Byung-Wook Cho

**Affiliations:** 1 Department of Animal Science, College of Natural Resources and Life Sciences, Pusan National University, Miryang 50463, Korea; 2 Division of Biochemistry, College of Medicine, Chungbuk National University, Cheong-Ju 28644, Korea; 3 Institute of Green-Bio Science and Technology, Seoul National University, Pyeongchang 25354, Korea; 4 Graduate School of International Agricultural Technology, Seoul National University, Pyeongchang 25354, Korea; 5 The Animal Molecular Genetics and Breeding Center, Jeonbuk National University, Jeonju 54896, Korea; 6 Department of Agricultural Convergence Technology, College of Agriculture and Life Sciences, Jeonbuk National University, Jeonju 54896, Korea

**Keywords:** Exercise Stress, Horse Fetal Muscle Cells, Peripheral Blood Mononuclear Cell Migration, Toll-like Receptor

## Abstract

**Objective:**

This study investigates the expression patterns of toll-like receptors (TLRs) and intracellular mediators in horse muscle cells after exercise, and the relationship between TLRS expression in stressed horse muscle cells and immune cell migration toward them.

**Methods:**

The expression patterns of the TLRs (*TLR2*, *TLR4*, and *TLR8*) and downstream signaling pathway-related genes (myeloid differentiation primary response 88 [*MYD88*]; activating transcription factor 3 [*ATF3*]) are examined in horse tissues, and horse peripheral blood mononuclear cells (PBMCs), polymorphonuclear cells (PMNs) and muscles in response to exercise, using the quantitative reverse transcription-polymerase chain reaction (qPCR). Expressions of chemokine receptor genes, i.e., C-X-C motif chemokine receptor 2 (*CXCR2*) and C-C motif chemokine receptor 5 (*CCR5*), are studied in PBMCs and PMNs. A horse muscle cell line is developed by transfecting SV-T antigen into fetal muscle cells, followed by examination of muscle-specific genes. Horse muscle cells are treated with stressors, i.e., cortisol, hydrogen peroxide (H_2_O_2_), and heat, to mimic stress conditions *in vitro*, and the expression of *TLR4* and *TLR8* are examined in stressed muscle cells, in addition to migration activity of PBMCs toward stressed muscle cells.

**Results:**

The qPCR revealed that *TLR4* message was expressed in cerebrum, cerebellum, thymus, lung, liver, kidney, and muscle, whereas *TLR8* expressed in thymus, lung, and kidney, while *TLR2* expressed in thymus, lung, and kidney. Expressions of TLRs, i.e., *TLR4* and *TLR8*, and mediators, i.e., *MYD88* and *ATF3*, were upregulated in muscle, PBMCs and PMNs in response to exercise. Expressions of *CXCR2* and *CCR5* were also upregulated in PBMCs and PMNs after exercise. In the muscle cell line, *TLR4* and *TLR8* expressions were upregulated when cells were treated with stressors such as cortisol, H_2_O_2_, and heat. Migration of PBMCs toward stressed muscle cells was increased by exercise and oxidative stresses, and combinations of these. Treatment with methylsulfonylmethane (MSM), an antioxidant on stressed muscle cells, reduced migration of PBMCs toward stressed muscle cells.

**Conclusion:**

In this study, we have successfully cultured horse skeletal muscle cells, isolated horse PBMCs, and established an *in vitro* system for studying stress-related gene expressions and function. Expression of *TLR4*, *TLR8*, *CXCR2*, and *CCR5* in horse muscle cells was higher in response to stressors such as cortisol, H_2_O_2_, and heat, or combinations of these. In addition, migration of PBMCs toward muscle cells was increased when muscle cells were under stress, but inhibition of reactive oxygen species by MSM modulated migratory activity of PBMCs to stressed muscle cells. Further study is necessary to investigate the biological function(s) of the TLR gene family in horse muscle cells.

## INTRODUCTION

The high adaptability of horses to exercise makes them a valuable model animal for studying the effects of exercise activity. Therefore, many studies of horses have focused on their physical and physiological adaptation to exercise, as well as the regulatory pathways involved and the mechanisms of targeted genes, while these exercise characteristics have helped with identifying the molecular mechanisms of adaptive responses to exercise [1].

Exercise induces various kinds of stress in muscles, including oxidative and inflammatory stress. Moreover, stress induced by exercise is considered one of the important external stimuli. Therefore, numerous exercise-related genes have been discovered through high-throughput analyses [2,3]. At the genetic level, stress response studies have been performed by analyzing differentially-expressed genes owing to stress [1,4] and have also been conducted on horse muscles [5–7].

Damage-associated molecular patterns (DAMPs) are important molecular signals that are released by damaged tissue in order to activate the immune system. Muscle tissue that has been damaged by acute injury secretes DAMPs to activate toll-like receptor (TLR) signaling, which in turn induces the expression of inflammatory genes for mediating tissue repair [8]. Subsequently, muscle cytokines and chemokines are released into the circulating plasma through activator protein 1 and the early phase of nuclear factor-kappa B transcription factors. These secreted cytokines and chemokines mediate the recruitment of monocytes from the bone marrow to the injury site. An atypical injury–regeneration event is characterized by the superimposition of the inflammatory response and recurring injury that perturbs the resolution of repair in the dystrophic muscle [9].

In our previous study, whole transcriptomes from blood and muscle tissues before and after exercise were analyzed by RNA sequencing, through which 32,361 UniGene clusters were identified. Among these, 1,305 differentially-expressed genes were discovered, many of which were enriched in various Gene Ontology terms, such as stress-related and immune-related genes, including genes coding TLRs and chemokine receptors [2]. In a previous study, we investigated the relationship between exercise stress stimuli and myokine genes in horse muscle cells [10]. Although our previous results had provided a valuable system for studying the function of exercise-related genes, the roles of the candidate genes remained unknown, due to the lack of a proper *in vitro* horse cell system for uncovering the mechanisms. Therefore, the present study was carried out to investigate the expressions of TLR and downstream signaling pathway-related genes in primary muscle cells derived from skeletal muscle of fetal Jeju horses, in order to determine whether exercise-related stress can modulate TLRs expressions in the muscle cell line and the migration of immune cells toward stressed horse cells. The results obtained will provide a valuable basis for studying the mechanism of exercise-induced muscle damage in horses.

## MATERIALS AND METHODS

### Animals

Three healthy horses (average age: 3.5 years old) were used in the study. All animal procedures used in the study were following conducted in compliance with international standards and were approved by the Institutional Animal Care and Use Committee of Pusan National University (Approval Number: PNU-2015-0864).

### Sample collection

Tissue samples were collected from biopsies of the cerebrum, thymus, lungs, liver, kidneys, and muscle of the slaughtered horses. Muscle and blood samples from each horse were collected before and after exercise (30 min). In brief, venous blood samples were collected using a 20 mL syringe and transferred to ethylenediaminetetraacetic acid (EDTA)-containing tubes. For the skeletal muscle biopsy, local anesthesia was administered to the gluteus medius, and a biopsy collection syringe was then used to obtain the muscle samples. All samples were stored at −80°C before RNA extraction.

### Isolation of peripheral blood mononuclear cells and polymorphonuclear cells

Peripheral blood mononuclear cells (PBMCs) and polymorphonuclear cells (PMNs) were isolated by the single-step centrifugation of whole-blood samples on a Polymorphprep column (Axis-Shield, Oslo, Norway) according to the manufacturer’s recommendations. The blood that was collected in the EDTA tube was layered over polymorphprep solution at a ratio of 1:1, and the tube column was then centrifuged at 500×g for 35 min. The PMN (granulocyte) and PBMC (lymphocyte and monocyte) layers were carefully collected and resuspended in 1× phosphate-buffered saline (PBS). After centrifugation of the suspensions at 14,000×g for 5 min, the supernatant was removed and the cell pellets were stored at −80°C until RNA extraction.

### Establishment of the horse muscle cell line

Simian vacuolating virus 40 T (SV40T) antigen was inserted into SBI’s PiggyBac Transposon System of cloning and expression vectors (System Biosciences, Palo Alto, CA, USA) through NotI digestion and ligation. The SV40T insertion site was then confirmed by DNA sequencing. In the PiggyBac cytomegalovirus (CMV)-SV40T-elongation factor 1 alpha (EF1α)-puromycin resistance (Puro) sequence, the CMV and EF1α promoter serve to control SV40T expression, while the puromycin resistance gene is used for transformant selection.

To establish the horse muscle tissue-derived cell line, the vector carrying both the PiggyBac transposase and CMV-SV 40T-EF1α-Puro sequences was transfected into the isolated muscle cells using Lipofectamine 3000 (Invitrogen, Carlsbad, CA, USA) according to the manufacturer’s protocol. Once the seeded cells had reached 80% confluency in the 6-well culture plates, they were washed with 1× PBS and replenished with 2 mL of culture medium without antibiotic–antimycotic. A plasmid DNA–lipid complex was added to each well, consisting of 7.5 μL of Lipofectamine 3000 reagent in 250 μL of Opti-MEM (Invitrogen, USA) and 10 μL of P3000 reagent, together with 2.5 μg of the PiggyBac transgene vector and PiggyBac transposase plasmid in 250 μL of Opti-MEM. At 1 day after lipofection, 10 μg/mL puromycin was added to select the cells that were stably transfected with the transgene. These puromycin-selected live cells, established as the muscle cell line, were further propagated and stored in liquid nitrogen.

### Horse muscle cell culture and *in vitro* stress-induced systems

The horse muscle cells were maintained and sub-passaged in medium 199 (Gibco, Grand Island, NY, USA) supplemented with 10% fetal bovine serum (FBS; Invitrogen, USA), 2% donor equine serum (DES; Hyclone, Carlsbad, CA, USA), and 1% antibiotic–antimycotic (Invitrogen, USA). The cells were cultured at 37°C in a humidified atmosphere with 5% CO_2_. Routine medium changes were performed three times a week. Cells at 70% to 80% confluency were gently washed twice with PBS and harvested using 0.05% trypsin-EDTA (Welgene, Gyeongsan-si, Korea) for expansion.

To induce various stresses, horse muscle cells at 70% to 80% confluency were incubated with the following stressors, i.e., 20 μg/mL cortisol or 600 μM hydrogen peroxide (H_2_O_2_) for 4 h. The cells were then incubated at 40°C for 1 h to induce heat stress, as previously described [11]. The muscle cells were also incubated with combined stressors to examine their combined effects.

### RNA extraction and complementary DNA synthesis

Total RNA was extracted from the horse PBMCs and PMNs using TRIzol reagent (Invitrogen, Karlsruhe, Germany) according to the manufacturer’s instructions. The purity of the extracted RNA was confirmed by measuring its absorbance at 230 and 260 nm using a spectrophotometer (ND-1000, Nanodrop Technologies Inc., Wilmington, DE, USA). RNA with a purity (OD230/260 nm value) of greater than 1.8 was selected for further analysis and stored at −80°C. To synthesize cDNA, 1 μg of RNA from each sample was reverse transcribed using the SuperScript III First-Strand Synthesis System (Invitrogen, Germany) according to the manufacturer’s instructions.

### Quantitative real-time polymerase chain reaction

To quantitate gene expression levels of TLRs, CCRs, and mediators in muscle tissues and blood cells before and after exercise, a quantitative real-time polymerase chain reaction (qRT-PCR) was conducted using the BioRad CFX-96 apparatus (BioRad, Hercules, CA, USA). PCR primer sequences are listed in Table 1. Each reaction was carried out in a 25 μL mixture containing 14 μL of SYBR Green Master Mix, 2 μL of forward primer (5 pmol), 2 μL of reverse primer (5 pmol), 5 μL of distilled water, and 2 μL (50 ng/μL) of cDNA. PCR conditions were as follows: a predenaturation step of 94°C for 5 min; 39 cycles of 94°C for 20 s, 56°C for 20 s, and 72°C for 30 s; and a final step of 72°C for 10 min. All measurements were performed in triplicate for all specimens, and the 2^−ΔΔCt^ method was used for comparing the data [12]. The relative expression of each target gene was calculated by normalizing the expression level against that of glyceraldehyde-3-phosphate dehydrogenase.

### Immune cell migration assay

For the immune cell migration assay, horse muscle cells were seeded into the lower chamber of a 24-well Corning Transwell plate and cultured after stress treatment according to the *in vitro* stress-induced systems described above. The media were then removed and PBMCs or PMNs (1×10^4^ cells per well) were seeded into the upper chamber of the Transwell plate. After culturing for 24 h, the cells in the lower chamber were collected and counted using the Muse Cell Analyzer (Merck Millipore, Darmstadt, Germany).

### Statistical analysis

Both T-test and analysis of variance statistical test were conducted to determine significance levels. Data were shown by mean±standard error.

## RESULTS

### Validation of toll-like receptor family genes, chemokines, and downstream signaling pathway-related genes during exercise stress induction

**Figure 1:**
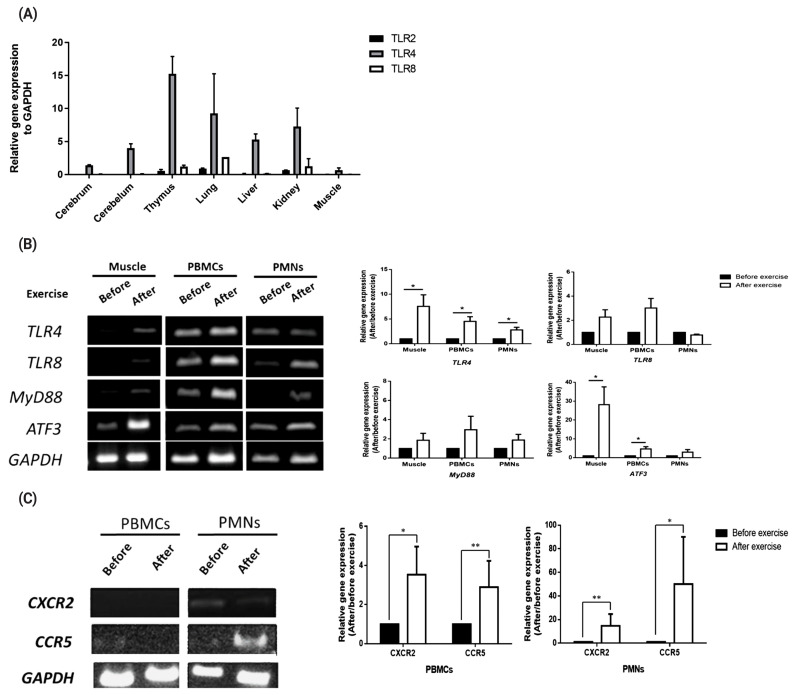
Expression of toll-like receptor family genes, chemokines, and downstream signaling pathway-related genes. (A) *TLR2*, *TLR4*, and *TLR8* expressions in various horse tissues. (B) *TLR4*, *TLR8*, *MYD88*, and *ATF3* expression levels in horse muscle biopsy samples and peripheral white blood cells (PBMCs and PMNs) before and after exercise. (C) *CXCR2* and *CCR5* expressions in muscle, PBMCs and PMNs before and after exercise. The relative expression of each gene was normalized to that of *GAPDH* and calculated with the 2^−ΔΔCT^ method (mean±standard error of the mean of N = 3; two-tailed student t-test). *TLR*, toll-like receptor; *MYD88*, MYD88 innate immune signal transduction adaptor; *ATF3*, activating transcription factor 3; *CXCR2*, C-X-C motif chemokine receptor 2; *CCR5*, C-C motif chemokine receptor 5; *GAPDH*, glyceraldehyde-3-phosphate dehydrogenase; PBMCs, peripheral blood mononuclear cells; PMNs, polymorphonuclear cells.

To validate transcript expression of *TLR2*, *TLR4*, and *TLR8* in various equine tissues, we conducted qRT-PCR with 7 tissues (cerebrum, cerebellum, thymus, lung, liver, kidney, and muscle). The results showed that *TLR4* was expressed in all the tissues studied, whereas *TLR8* was expressed only in the thymus, lung, and kidney tissues and at a lower level than *TLR4* (Figure 1A). *TLR4* and *TLR8* gene expression levels in muscle cells, PBMCs, and PMNs before and after exercise were further analyzed by RT-PCR (Figure 1B). Also transcript expression of these two TLRs and the signaling molecules, i.e., myeloid differentiation primary response 88 (MYD88) and activating transcription factor 3 (ATF3) were quantitated by qRT-PCR. The results showed that expression of TLR4 was significantly increased after exercise (p<0.05), while that of TLR8 and MYD88 was increased but statistically not significant (p>0.05, Figure 1B). As the negative regulator of TLR signaling, ATF3 was significantly increased in the muscle tissue and PBMCs (Figure 1B). The results showed that expressions of C-X-C motif chemokine receptor 2 (CXCR2) and C-C motif chemokine receptor 5 (CCR5) were lower in PBMCs but higher in PMNs (especially post-exercise for CCR5) (Figure 1C). According to the qPCR data, CXCR2 and CCR5 expression levels tended to increase after exercise (p<0.05), but the difference relative to the levels before exercise was not statistically significant (p>0.05, Figure 1C).

### Establishment of horse muscle derived muscle cell-line

**Figure 2:**
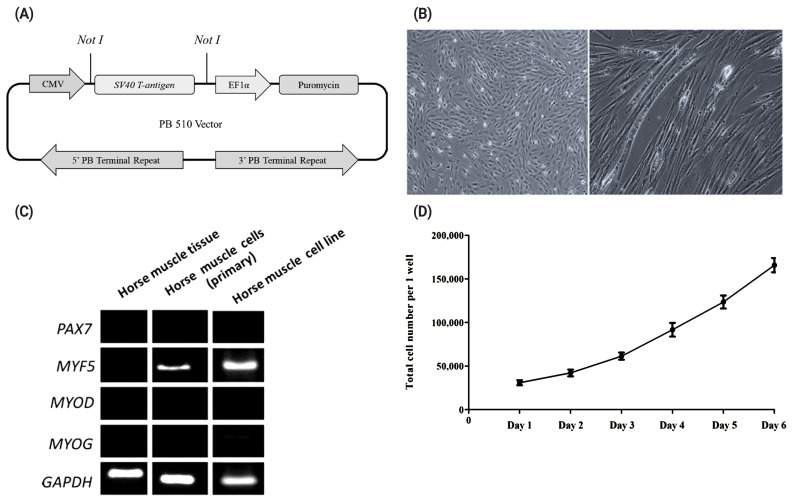
Immortalized horse muscle cells and expressions of myogenic markers in the horse muscle tissue, primary muscle cells, and muscle cell line. (A) Schematic of the PiggyBac transposase plus CMV-SV40T-EF1α-Puro vector construction. (B) Morphology of horse muscle cells and differentiated horse muscle cells (magnification, 200×). (C) Real-time polymerase chain reaction analysis of myogenic markers in horse muscle tissue, primary horse muscle cells, and SV40T antigen-transfected horse muscle cells. (D) Proliferation analysis of undifferentiated horse muscle cells. CMV, cytomegalovirus; SV40T, simian vacuolating virus 40 T; EF1α, elongation factor 1 alpha; Puro, puromycin; *PAX7*, paired box 7; *MYF5*, myogenic factor 5; *MYOD*, myogenic differentiation; *MYOG*, myogenin; *GAPDH*, glyceraldehyde-3-phosphate dehydrogenase.

To establish a horse muscle cell line, skeletal muscle from a neonatal Thoroughbred and fetus of Jeju pony were transfected with an SV40T antigen-carrying vector. PiggyBac transposon-mediated SV40T expression vector (piggyBac CMV-SV40T-EF1α-puromycin) was designed and constructed (Figure 2A) using simian virus 40 (SV40) to immortalize the cells. Following transfection of the piggyBac CMV-SV40T expression vector system, transfected cells were selected with puromycin.

Horse muscle derived cells were maintained in Medium 199 supplemented with 10% FBS and 2% DES (Figure 2B) and a growth curve of these cells is shown in Figure 2D. Next, to determine whether the cell lines developed after selection maintained the features of muscle cells, we performed RT-PCR for myogenic markers (Figure 2C). As results, paired box 7 (PAX7), myogenic differentiation (MyoD), and myogenin (MyoG) were weakl, but the other myogenic marker, such as myogenic factor 5 (Myf5) was expressed in a muscle cell line compare to primary horse muscle cells. Subsequently, a horse cell line was subjected to differentiation to confirm the capacity for differentiation into myotube cells. Horse muscle cells with 80% confluence were cultured in Medium 199 supplemented with 2% FBS for 12 days. Myoblasts were fused into multi-nucleated fibers (Figure 2B), but it is of note that horse muscle cells takes longer time to differentiate than those of mouse muscle cell line C2C12, immortalized mouse myoblast cell line.

### Expression patterns of TLR4, TLR8, chemokines, and downstream signaling pathway-related genes in horse muscle cells under stress conditions

**Figure 3:**
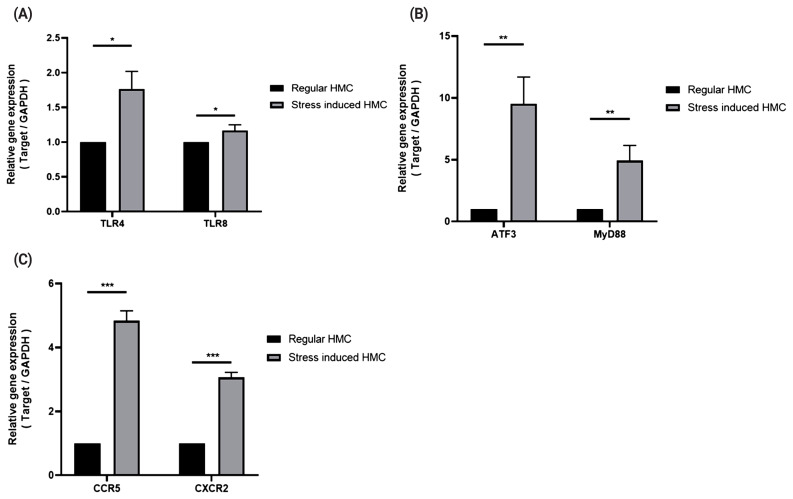
Expression patterns of *TLR4*, *TLR8*, chemokine receptors, signaling mediators in horse muscle cells under stress conditions. Real-time polymerase chain reactions were performed to measure gene expression levels of TLR4 and TLR8 (A), signaling mediators (B), and chemokine receptors (C). The relative expression for each gene was normalized to that of *GAPDH* and calculated with the 2^−ΔΔCT^ method (mean±standard deviation of triplicate experiments; two-tailed student t-test). HMC, horse muscle cells; PBMCs, peripheral blood mononuclear cells; *TLR*, toll-like receptor; *MYD88*, myeloid differentiation primary response 88; *ATF3*, activating transcription factor 3; *GAPDH*, glyceraldehyde-3-phosphate dehydrogenase.

To induce stresses caused by exercise, we treated horse muscle cells with cortisol, H_2_O_2_, and heat stress, or in combination, to reproduce stresses caused by exercise [13] *in vitro*, and determined the expressions of TLRs (TLR4 and TLR8), chemokine receptors (CXCR2 and CCR5), and signaling mediators (ATF3 and MYD88) in the stressed muscle cells. Both *TLR4* and *TLR8* showed increased mRNA expression under stress conditions (Figure 3A; p<0.5), and expressions of *CXCR2*, *CCR5*, *ATF3*, and *MYD88* under stress conditions were observed to be over three times higher than those under non-stimulated conditions (Figure 3B; p<0.05, 3C; p<0.005).

### PBMC migration to stress induced muscle cells, and effect of methyl sulfonyl methane on stress induced PBMC migration

**Figure 4:**
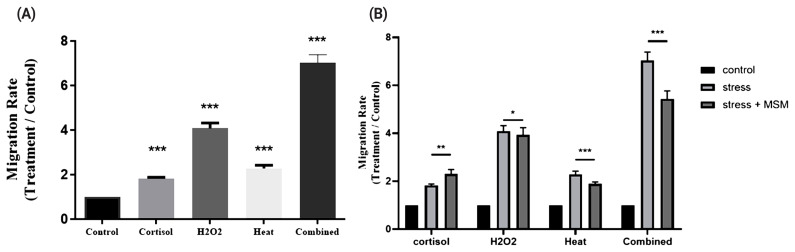
Migration of PBMCs to stressed horse muscle cells and effect of MSM on this. (A) Rate of PBMCs migration from upper to lower chamber following stress stimulation. (B) Rate of PBMCs migration to stressed horse muscle cells in the presence of MSM. PBMCs, peripheral blood mononuclear cells; MSM, methylsulfonylmethane. n = 3; ** p<0.05; *** p<0.005.

To investigate the effects of stressed muscle cells on the migration capacity of immune cells, transwell assays were conducted to evaluate the migration of PBMCs or PMNs toward muscle cells under various stress conditions. PBMC migrated more to the muscle cells which were under combined stresses, compared to control muscle cells (p<0.05, Figure 4A). In addition, we investigated the effect of methyl sulfonyl methane (MSM) on the migration of PBMCs toward stressed muscle cells by transwell assay, as described above (Figure 4B).

## DISCUSSION

Numerous cytokines are secreted in muscle after exercise to recover injured muscle [14,15]. The DAMPs are released upon cellular stress or tissue injury and DAMPs initiate the production of inflammatory cytokines and chemokines to initiate inflammatory responses [16]. Despite cytokines represent the key initiators of inflammation and horse is superior model for exercise, cytokine and muscle recover researches are poorly studied in horse. DAMPs are released from the extracellular or intracellular space by tissue injury or cell death [17]. These DAMPs are recognized by macrophages, and inflammatory responses are triggered by different pathways, including TLRs and inflammasomes [17,18]. Activated TLRs by DAMPs induced expression of inflammatory genes to mediate tissue repair [8].

In this study, we examined the expression pattern of TLRs, signaling molecules, and chemokines by exercise (Figure 1). The result shown TLR family genes, chemokines, and downstream signaling pathway-related genes increased after exercise. Taken together, these results demonstrate that TLR4 was upregulated in both muscle tissue and white blood cells after exercise-induced stress. These results are consistent with those of other studies that have also shown increased expression of TLR4 [19] and ATF3 in mice following exercise, suggesting that upregulation of TLR signaling induces expression of the negative signaling factor ATF3 to maintain homeostasis of muscle injury and recovery [20]. In addition, we examined chemokine receptor expression by exercise. Result shows that CXCR2 and CCR5 increased after exercise. It is assumed that horse white blood cells may respond directly to their ligands (presumably DAMPs) released from muscle cells, although this single time point could not clearly show any difference between the effects before or after exercise.

In this study, we established a horse muscle cell line transfected with an SV40T antigen-carrying vector (Figure 2A, 2B), and to determine whether the cells we obtained matched muscle cells, we performed RT-PCR of myogenic markers (Figure 2C). Myogenic regulatory factors (MRFs) induced muscle differentiation, and expressing muscle specific markers [21]. MRFs include a group of four protein; MyoD, Myf5, MyoG, and MRF4 [22]. Through expression of Myf5, it was reasonable muscle derived cells are myogenic cells, however, muscle cells are heterogeneous, therefore further study is required to isolate myoblast.

In addition, we validates expression of TLR family genes, chemokine receptors, and signaling mediators in horse muscle cells under stress conditions (Figure 3). Several studies have also shown that expression of the *TLR4* gene was increased under oxidative stress [23,24] and that heat stress can induce TLR2 and TLR4 via the p38 kinase signaling pathway [25,26]. It is assumed that TLRs induce the innate immune response through activation of the signal transduction cascade via TIR domain-containing adapters such as MYD88 or TIR domain containing adaptor protein [27,28]. ATF3, a member of the ATF/cyclic adenosine monophosphate response element-binding protein family of bZIP transcription factors, binds to the consensus cyclic adenosine monophosphate response element site, where it functions as a transcriptional repressor by forming a homodimer. In macrophages from human PBMCs, ATF3 mRNA expression is increased by lipopolysaccharide (a TLR4 ligand), the Bacillus Calmette-Guérin vaccine, and interferon. Moreover, ATF3 mRNA expression is upregulated by TLR4 signaling and is part of the negative feedback loop that regulates the lipopolysaccharide-stimulated inflammatory response [29]. ATF3 is also one of the immediate-early response genes [30–33] and is induced by various physiological and pathological stimuli, including anticancer drugs [34], proteasome inhibitors [35], growth-stimulating factors like serum [36,37], and esophageal cancer cells [38]. It is thus reasonable to suggest that both ATF3 and MYD88 function in cellular stress responses.

Although we did not examine the molecular mechanisms for up-regulation of TLRs, chemokine receptors and signaling mediators genes in this study, it is reasonable to assume that DAMPs released from muscle or muscle cells damaged by stressors may activate the cellular pathways leading to upregulation of TLRs and others genes, that were studied here, either sequentially or simultaneously, as a previous study has reported [8]. Therefore, the molecular mechanisms for the up-regulation by DAMPs of TLRs and others in horse muscle cells need to be elucidated in a future study.

Finally, we evaluate the migration of PBMCs or PMNs toward muscle cells under various stress conditions, and effect of MSM on migration of PBMCs toward stressed muscle cells (Figure 4). Result shows that PBMC migrated more to the muscle cells which were under combined stresses. It is known that chemokines recruit immune cells to damaged tissues [39–41] or including injured muscle [42]. Taken together, it is assumed that injured muscle cell secrets chemokine to recruit PBMC to recover damage through TLRs. In addition, we found that migration of PBMCs toward the stressed muscle cells was reduced in the presence of MSM except in the cortisol group, supporting the notion that MSM can reduce stress, in turn leading to a reduction in PBMCs migration. It remains unclear, however, how MSM modulates cellular pathways of stressed muscle cells, resulting in a reduction in PBMCs migration. A previous study has reported that MSM reduces cortisol-induced stress by modulating the expression of succinate dehydrogenase complex flavoprotein subunit A/hypoxanthine phosphoribosyl-transferase 1, which was governed by p53 in racehorse muscle [43]. Further study is required to delineate the molecular pathways in horse muscle cells that lead to reduced migration of PBMCs by MSM.

## CONCLUSION

This study investigated the expressions of TLRs, chemokine receptors, and signaling mediators genes in horse muscle and blood cells in response to exercise, and further studied their expressions in cultured muscle cells under stress. Exercise and cellular stresses induced by hormone, ROS, and heat, or combinations of these, in muscle tissue or muscle cells increased expressions of these genes, and also increased migration of PBMCs to stressed muscle cells. Treatment with an antioxidant reduced stress-induced migration of PBMCs to muscle. Further study is necessary, however, to uncover the biological function(s) of the TLR family of genes in horse muscle cells. This study is intended to provide a cellular system that can be used for deciphering the molecular mechanisms of cellular stress response in horses, and to enable the evaluation of agents for alleviating it.

## Figures and Tables

**Table 1:** Primer sets used in this study

Primer name	Primer sequence (5’ to 3’)	Tm (°C)	Product size (bp)
PAX7 F	GACTCCCGATGTGGAGAAAA		
PAX7 R	GCTTCGTCCTCCTCCTCTTT	58	434
MyoD F	CAAGCGCAAGACCACTAACG		
MyoD R	AGGTGCCATCGTAGCAGTTC	58	386
MyoG F	GCGTGCAAGGTGTGTAAGAG		
MyoG R	TGTCCACAATGGAGGTGAGC	58	415
Myf5 F	TTTCGGGGACGAGTTTGAGC		
Myf5 R	CCAGACGGGGCTGTTACATT	58	445
TLR4 F	GGCATCATCTTCATCGTCCT		
TLR4 R	CAAGGCTTTCCTGAGTCGTC	60	153
TLR8 F	GTGCAGGAAATAGCGTCTGG		
TLR8 R	CTCATCAAGGGCCGGAAATG	60	206
ATF3 F	CCATCCAGAACAAGCACCTC		
ATF3 R	GCATTCACACTCTCCAGCTTC	60	235
MyD88 F	AGGATGGTGGTGGTTGTCTC		
MyD88 R	AGGATGCTGGGGAACTCTTT	60	152
CCR5 F	CAGAAAACCGACGTGAGACA		
CCR5 R	GGGAGGGTGAGAAGGAAAAG	60	192
CXCR2 F	ATGCCCTGGTCGTCATCTAC		
CXCR2 R	GTCAAGGCAAAGAGCAGGTC	62	154
GAPDH F	GGTGAAGGTCGGAGTAAACG		
GAPDH R	AATGAAGGGGTCATTGATGG	60	106

PAX7, paired box 7; MyoD, myogenic differentiation; MyoG, myogenin; Myf5, myogenic factor 5; TLR, toll-like receptor; ATF3, activating transcription factor 3; MyD88, MYD88 innate immune signal transduction adaptor; CCR5, C-C motif chemokine receptor 5; CXCR2, C-X-C motif chemokine receptor 2; GAPDH, glyceraldehyde-3-phosphate dehydrogenase.
